# Supplementary material for: Integration of palliative care into phase I oncology trials: A qualitative interview study with patients, informal caregivers, and healthcare providers
Source: PLoS One. 2026 May 5;21(5):e0347349. doi: 10.1371/journal.pone.0347349 (PMC13143114; doi:10.1371/journal.pone.0347349)
Supplement: S2 File — Topic guides. (DOCX) [file pone.0347349.s002.docx]

**S2 Topic guides**

**Interview Topic Guide – Patients**

**1. Background**

- Can you describe your cancer treatment journey so far?

**2. Decision-Making and Information**

- How was your conversation with the oncologist before starting the Phase I study?
- How were you invited to participate? Who initiated the conversation?
- What information were you given about the study (e.g., risks, side effects, chances of success)?
- Was the information clear and understandable?
- What made you decide to participate?
- What were your main considerations? Did you weigh pros and cons?
- Did you discuss the decision with others? How did that influence you?
- What do you hope to achieve by participating?

**3. Quality of Life**

- How has the Phase I treatment been for you so far?
- Have you experienced any side effects or burdens (physical, emotional, social)?
- Have there been any positive aspects of participating?
- What is important to you in life? What brings you joy?
- What does a good day or a bad day look like for you?
- Has the study affected your quality of life? In what ways?
- Are the things that matter to you discussed with your care team?
- Who usually initiates these conversations—you or the care providers?
- How would you like these topics to be addressed during the study?
- Do you feel enough attention is paid to what matters to you? If not, what would help?

**4. Palliative Care**

- What comes to mind when you hear the term “palliative care”?
- Have you heard about palliative care before? From whom?
- [Provide definition and overview of palliative care domains: physical, psychological, social, existential, communication, coordination.]
- Have any of these aspects been addressed by your care team?
  - Before the Phase I study?
  - During the study?
- If yes:
  - When and by whom?
  - Were you prepared for the conversation?
  - How did you feel about it?
  - What went well or could have been better?
- If no:
  - Would you have liked to discuss these topics? Why or why not?
  - What would it mean to you to receive this kind of support?
  - Are there specific domains you find important in this context?
  - Have you noticed a change in attention to palliative care since joining the study?
- What do you see as potential benefits or drawbacks of palliative care in this context?

**5. Advance Care Planning**

- How do you view your current treatment and illness trajectory?
- Are you satisfied with your care? Are there things you would like to change?
- Have you thought about what you would want if the treatment doesn’t work?
- Have you discussed this with anyone? What would you want or not want in that case?

**6. Closing**

- What should we definitely remember from this conversation?
- Do you have any remaining concerns or questions?

**Interview Topic Guide – Family Caregivers**

**1. Background and Role**

- What is your relationship to the patient?
- What is your role in their care?
- Are you involved in the medical aspects of their care?
- When and how did you take on this role?

**2. Decision-Making and Information**

- How was the decision made to participate in the Phase I study?
- Were you present at the conversation about participation?
- What information was shared, and how did you perceive it?
- Did the patient have doubts? Did you discuss the decision together?
- How do you feel about the patient’s participation?
- What are your expectations, and what do you think the patient expects?

**3. Quality of Life**

- What do you think is important to the patient in life?
- What does a good or bad day look like for them?
- How has the study affected their quality of life?
- Has their perception of quality of life changed during the illness or since joining the study?
- Are these topics discussed with healthcare providers?
- Do you feel enough attention is paid to quality of life?
- How does this affect your role as a caregiver?
- Have there been moments when you wanted to discuss quality of life with providers?

**4. Palliative Care**

- What comes to mind when you hear the term “palliative care”?
- Where or from whom have you heard about it?
- [Provide definition and overview of palliative care domains: physical, psychological, social, existential, communication, coordination.]
- Has palliative care been addressed by healthcare providers?
  - Before the Phase I study?
  - During the study?
- Has the patient received palliative care, to your knowledge?
- What went well or less well?
- Would you have liked to discuss palliative care? With whom?
- How could it support you as a caregiver?
- Have you noticed a change in attention to palliative care since the study began?
- What do you see as the benefits or drawbacks of palliative care in this context?
- Which aspects of palliative care do you find most important?
- Have you ever discussed palliative care needs with the patient?
- Would you like more support in having these conversations?

**5. Advance Care Planning**

- How do you view the patient’s current treatment and illness trajectory?
- Do you know what the patient would want if the treatment is no longer effective?
- Have you or the patient thought or talked about this?
- Has this been discussed with healthcare providers?
- Have you wanted to bring it up? Why or why not?

**6. Closing**

- What should we definitely remember from this conversation?
- Do you have any remaining concerns or questions?

**Interview Topic Guide – Phase I Staff**

**1. Study Population**

- What does the patient population in your studies typically look like?
- What types of cancer are most commonly studied?
- Are there shared characteristics among participants?

**2. Recruitment**

- How do you recruit patients for your studies?
- What methods or protocols are used?
- What challenges or barriers do you encounter?
- How is collaboration managed during referral and after study completion?

**3. Quality of Life**

- Is sufficient attention paid to patients’ quality of life alongside study outcomes?
- What does “quality of life” mean to you in this context?
- How is it addressed in your practice?
- What improvements could be made?
- In your view, does participation in a Phase I study affect patients’ quality of life?
- If so, in what ways?

**4. Palliative Care**

- What is your understanding of palliative care?
- [Provide definition and overview of palliative care domains: physical, psychological, social, existential, communication, coordination.]
- What role does palliative care currently play in your studies?
- Do you discuss palliative care with participants?
  - If yes: when and how? How is it received?
  - If no: who does, and how aware are you of these discussions?
- Do any of your participants receive palliative care?
  - If yes: how often, and what is the impact?
  - If no: do you see a need for it?
- What is your view on systematically integrating palliative care into Phase I studies?
  - What factors influence this (e.g., patient, physician, cancer type)?
- How do you envision this integration in practice?
  - Which aspects of palliative care are most important?
  - What are the barriers and facilitators?
  - Who should be responsible for implementation?
  - What organizational or financial changes would be needed?

**5. Advance Care Planning**

- Would it be valuable for Phase I patients to discuss care preferences in case the treatment is ineffective?
- Is this discussed by the oncologist? How is that information shared with you?
- If not, do you take on this role? Who should?
- Do patients or families initiate these conversations?
- Are there sufficient opportunities to discuss these topics during consultations?
- When would be the ideal time to address them?
- What conditions are needed to have these conversations effectively?

**6. Closing**

- What should we definitely remember from this conversation?
- Do you have any remaining concerns or questions?

**Interview Topic Guide – Oncologists**

**1. Experience with Phase I Trials**

- What is your understanding of Phase I studies?
- What are the pros and cons of participation for patients?
- Why do patients choose to participate?

**2. Role and Communication**

- How do you engage with Phase I studies in your role?
- How is communication and collaboration with Phase I teams organized?
- Is there a handover when a patient starts a Phase I study?
- Do you remain involved during and after the study?
- Is there continued contact with the patient or their psychologist?

**3. Referral and Decision-Making**

- Do you refer patients to Phase I studies?
- How often, and how do you present this option?
- How do patients otherwise come into contact with Phase I studies?
- What are the pros and cons of referring patients?
- How are you informed about ongoing Phase I studies?
- Is this coordinated within your department or individually?

**4. Discussing Participation**

- How is potential participation discussed with patients?
- Do you decide alone or in a team?
- What factors influence the decision?
- At what point in the care trajectory is this offered?

**5. Quality of Life**

- What does “quality of life” mean to you as a care provider?
- Does participation in Phase I studies affect patients’ quality of life?
- Is enough attention paid to quality of life during the study?
- How is this addressed (e.g., physical, psychological, social, existential aspects)?
- What improvements are needed?

**6. Palliative Care**

- What is your understanding of palliative care?
- [Provide definition and overview of palliative care domains: physical, psychological, social, existential, communication, coordination.]
- Do your Phase I patients receive palliative care?
- How does this compare to non-Phase I patients?
- Do you discuss palliative care with patients?
  - When and how?
  - Do you use the term “palliative care”?
  - How do patients respond?
- What is the impact of palliative care during the study?
- Is enough attention paid to palliative care in Phase I settings?
- What are your thoughts on integrating palliative care systematically?
  - What factors influence this (e.g., patient, physician, cancer type)?
  - Is integration feasible? What barriers or facilitators exist?
  - What is your role as an oncologist?

**7. Advance Care Planning**

- Would it be valuable to discuss care preferences if the treatment proves ineffective?
- Is this discussed by the Phase I team?
- If not, do you take on this role? Who should?
- Do patients or families initiate these conversations?
- Are there enough opportunities to discuss this during consultations?
- When should these conversations ideally take place?
- What are the conditions for having these discussions effectively?

**8. Closing**

- What should we definitely remember from this conversation?
- Do you have any remaining concerns or questions?

**Interview Topic Guide – Palliative Care Providers**

**1. Background and Role**

- In what ways do you engage with patients with cancer?
- At what stage of treatment are you typically involved?
- What does “quality of life” mean to you as a care provider?
- What are the most important aspects of palliative care in your view?

**2. Experience with Phase I Patients**

- What do you know about Phase I studies?
- Have you been involved in the care of patients participating in Phase I trials?
  - In what setting, and what kind of care did you provide?
  - How long do patients typically remain in Phase I treatment?
  - How did you experience the timing of your involvement?
  - What other professionals are involved, and with whom do you collaborate most?
  - What kind of care do you mainly provide?
  - How important is quality of life for these patients?
- If not involved: What would be needed to involve palliative care more systematically in Phase I settings?

**3. Perceived Benefits and Challenges**

- What are the potential benefits and/or drawbacks of Phase I trial participation for patients and families?
- Why do you think patients choose to participate?
- What role do you see for palliative care in Phase I studies?
  - What added value could it offer?
  - Would you approach these patients differently than others? Why or why not?
  - What factors influence the role of palliative care in this context?
  - Who should initiate palliative care discussions—specialist or generalist providers?

**4. Integration of Palliative Care**

- What is your view on systematically integrating palliative care into Phase I studies?
  - What factors influence this (e.g., patient, physician, cancer type)?
- How do you envision this integration in practice?
  - Which aspects of palliative care are most important to include?
  - Is integration feasible?
  - What barriers or facilitators exist?
  - Who should take the lead in facilitating integration?
  - What organizational or financial changes would be needed?

**5. Advance Care Planning**

- Would it be valuable for Phase I patients to discuss what matters to them or which (palliative) treatments are possible if the study treatment is ineffective?
  - Who should lead these conversations?
  - When should they ideally take place?
  - What are the conditions for having these conversations effectively?

**6. Closing**

- What should we definitely remember from this conversation?
- Do you have any remaining concerns or questions?

**Interview Topic Guide – General Practitioners**

**1. Background and Role**

- How do you encounter patients with cancer in your practice?
- Are they long-term patients or newly referred?

**2. Experience with Phase I Studies**

- What do you know about Phase I studies?
- What are the potential benefits and drawbacks of participation?
- Have you referred patients to Phase I studies?
  - How did that process go?
  - What role did you play in informing or supporting the patient?
  - Was it clear what steps to take?
- Have you been contacted by Phase I teams for referral or follow-up?
  - If yes: how was that communication?
  - If no: would you like to be involved? In what way?
- How would you like to be involved in the follow-up of these patients?
  - What role can GPs play in supporting them?
  - What are the conditions for meaningful involvement?
  - What are the barriers and facilitators?

**3. Quality of Life**

- How does participation in a Phase I study affect patients’ quality of life?
- What is needed to ensure continued attention to quality of life?
  - Who should be responsible?
  - What are current gaps or challenges?

**4. Palliative Care**

- What is your understanding of palliative care?
- [Provide definition and overview of palliative care domains: physical, psychological, social, existential, communication, coordination.]
- What are the most important aspects of palliative care in your view?
- How do you address palliative care in your practice?
  - When do you bring it up?
  - What challenges do you face?
- Who should initiate palliative care for Phase I patients?
  - Specialist or generalist care?
  - What factors influence this?
- What is your view on systematically integrating palliative care into Phase I studies?
  - What factors influence this (e.g., patient, physician, cancer type)?
- How do you envision this integration in practice?
  - Which aspects are most important?
  - What are the barriers and facilitators?
  - Who should take the lead?
  - What organizational or financial changes are needed?
- What could GPs contribute to palliative care in this context?
  - What should remain the responsibility of specialist teams?
  - What support do you need?

**5. Advance Care Planning**

- Would it be valuable for Phase I patients to discuss what matters to them or which (palliative) treatments are possible if the study treatment is ineffective?
  - Do you see a role for GPs in this?
  - When should these conversations ideally take place?
  - What are the conditions for having these conversations effectively?

**6. Closing**

- What should we definitely remember from this conversation?
- Do you have any remaining concerns or questions?
