# Supplementary material for: Integration of palliative care into phase I oncology trials: A qualitative interview study with patients, informal caregivers, and healthcare providers
Source: PLoS One. 2026 May 5;21(5):e0347349. doi: 10.1371/journal.pone.0347349 (PMC13143114; doi:10.1371/journal.pone.0347349)
Supplement: Attachment — Submitted filename: Response to Reviewers.docx [file pone.0347349.s004.docx]

**RESPONSE TO REVIEWERS’ COMMENTS**

**MANUSCRIPT ID: PONE-D-25-38276**

**Integration of palliative care into phase I oncology trials: A qualitative interview study with patients, informal caregivers, and healthcare providers**

We thank the editor for the opportunity to respond to comments and submit a revision of the manuscript. We also thank the reviewer for their thorough and helpful feedback to help us improve the manuscript, and address their comments below.

All modifications to the manuscript described below have been made in the version titled ‘Revised Manuscript with Track Changes’.

A clean version without tracked changes has also been uploaded as a separate file labeled ‘Manuscript’. Please note that the noted locations of changes in the table below refer to the lines in the clean version of the Manuscript.

| Comments Editor: | Response to the editor’s comments | Location of changes |
| --- | --- | --- |
| 1. Please ensure that your manuscript meets PLOS ONE's style requirements, including those for file naming. The PLOS ONE style templates can be found at <https://journals.plos.org/plosone/s/file?id=wjVg/PLOSOne_formatting_sample_main_body.pdf> and <https://journals.plos.org/plosone/s/file?id=ba62/PLOSOne_formatting_sample_title_authors_affiliations.pdf> | We adapted the heading font sizes and file descriptions to be in line with PLOS ONE’s stye requirements. | Throughout manuscript |
| 2. We note that you have indicated that there are restrictions to data sharing for this study. For studies involving human research participant data or other sensitive data, we encourage authors to share de-identified or anonymized data. However, when data cannot be publicly shared for ethical reasons, we allow authors to make their data sets available upon request. For information on unacceptable data access restrictions, please see <http://journals.plos.org/plosone/s/data-availability#loc-unacceptable-data-access-restrictions>.  Before we proceed with your manuscript, please address the following prompts:  a) If there are ethical or legal restrictions on sharing a de-identified data set, please explain them in detail (e.g., data contain potentially identifying or sensitive patient information, data are owned by a third-party organization, etc.) and who has imposed them (e.g., a Research Ethics Committee or Institutional Review Board, etc.). Please also provide contact information for a data access committee, ethics committee, or other institutional body to which data requests may be sent.  b) If there are no restrictions, please upload the minimal anonymized data set necessary to replicate your study findings to a stable, public repository and provide us with the relevant URLs, DOIs, or accession numbers. Please see <http://www.bmj.com/content/340/bmj.c181.long> for guidelines on how to de-identify and prepare clinical data for publication. For a list of recommended repositories, please see <https://journals.plos.org/plosone/s/recommended-repositories>. You also have the option of uploading the data as Supporting Information files, but we would recommend depositing data directly to a data repository if possible.  Please update your Data Availability statement in the submission form accordingly. | Our study is based on qualitative interviews with patients, family caregivers, and healthcare professionals. The interview transcripts contain potentially identifying and sensitive personal information that cannot be fully anonymized without compromising participant confidentiality. According to the approval conditions set by the Medical Ethics Committee of UZ Brussel/VUB, full transcripts may not be shared publicly.  However, pseudonymized excerpts relevant to the study can be shared upon reasonable request. Any such request will be reviewed by the research team and must receive approval from the Ethics Committee before data access is granted.  We have therefore updated our Data Availability Statement as follows:  ***Revised Data Availability Statement***  *The data underlying this study consist of qualitative interview transcripts containing potentially identifying and sensitive information from patients, family caregivers, and healthcare professionals. In accordance with the approval conditions of the Medical Ethics Committee of UZ Brussel/VUB, these data cannot be shared publicly to protect participant confidentiality.*  *Pseudonymized excerpts relevant to the findings can be shared upon reasonable request. Data requests may be sent to either the first author or the Medical Ethics Committee of UZ Brussel/VUB (*[*Ethiek@uzbrussel.be*](mailto:Ethiek@uzbrussel.be)*). Any data sharing will only occur after formal approval from the Ethics Committee and in accordance with applicable data protection regulations.* | Data availability statement |
| 3. In the online submission form, you indicated that as the data concern interview transcripts with participants, including patients, family caregivers, and healthcare professionals, and as they as such contain personal data, they cannot be shared publicly. Requests for pseudonymized versions of the data may be addressed to the first author. Every request will be evaluated on an individual basis and the ethics committee of the Vrije Universiteit Brussels will be contacted for approval before any sharing of participant-level data.  All PLOS journals now require all data underlying the findings described in their manuscript to be freely available to other researchers, either 1. In a public repository, 2. Within the manuscript itself, or 3. Uploaded as supplementary information. This policy applies to all data except where public deposition would breach compliance with the protocol approved by your research ethics board. If your data cannot be made publicly available for ethical or legal reasons (e.g., public availability would compromise patient privacy), please explain your reasons on resubmission and your exemption request will be escalated for approval. | As described in our revised Data Availability Statement (see response to comment 2), the data for this study consist of qualitative interview transcripts containing potentially identifying and sensitive personal information from patients, family caregivers, and healthcare professionals. In accordance with the approval conditions set by the Medical Ethics Committee of UZ Brussel/VUB, these data cannot be made publicly available without violating participant confidentiality and the terms of ethical approval. Pseudonymizing the full transcripts to remove all identifying information is not possible given the context of the study in which relevant references to clinical experiences are made.  We therefore request an exemption from public data deposition on ethical grounds. Pseudonymized excerpts relevant to the study findings can, however, be made available upon reasonable request. Any such request will be reviewed by the research team and must receive prior approval from the Medical Ethics Committee of UZ Brussel/VUB before data access is granted.  The revised Data Availability Statement in our manuscript and submission form reflects this policy and provides the relevant institutional contact details. | Data availability statement |
| 4. If the reviewer comments include a recommendation to cite specific previously published works, please review and evaluate these publications to determine whether they are relevant and should be cited. There is no requirement to cite these works unless the editor has indicated otherwise. | Not applicable. | N/A |
| Comments Reviewer 1 | Response to the reviewer’s comment | Location of changes |
| I would like to thank the authors for their initiative. It is important to assess the justification for using cancer patients with non-curative type with limited overall survival in Phase 1 trials. I would like to however, raise few points, and request the authors to address them, if possible.  I have mentioned these doubts in details in the manuscript as side notes.  Overall, the study has merits to add to the better knowledge of patient perspectives which are currently missing in healthcare many a times. Using them can make treatment and research patient-centric. | We thank the reviewer for their appreciation for our study, as well as for taking the time to provide very thorough feedback on our manuscript, which we have used to improve our manuscript. Below, we reply point-by-point to the comments made by the reviewer in their review. As the reviewer mentions, they have also added detail-level comments as notes to our manuscript as well – since the reviewer summarized these in their review, we reply to the latter formulations rather than to the detail-level comments as notes to our manuscript. Where useful for clarity, we have copied these detail-level comments into our responses. | N/A |
| 1) Is there any method such as qualitative interview study? Most qualitative studies use interviews as a data collection tool. | We use the wording ‘qualitative interview study’ in the title to succinctly note that our study was of a qualitative nature and that we indeed used interviews as a data collection tool. We believe this is important to note, as while indeed many qualitative studies use interviews, not all do (e.g. studies analyzing observational data), and as interviews can also be a method to collect quantitative data (e.g. surveys). | N/A |
| 2) The authors can comment on the justification as available in literature to involve patients with such advanced stage in phase1 trials. They may briefly discuss the current scenario of PC in Belgium in general and the problems faced by the trial participants to indicate the current gaps.  -- The reviewer clarifies this comment further by noting in the manuscript “What is the justification to involve patients with a overall survival of one year to be involved in phase 1 trials assessing drug dosage, instead of proper PC and initiation of EoLC?”, as well as “elaborate the current scenario of PC in Belgium in general and the problems faced by the trial participants”. | We would like to note that we agree with the reviewer questioning the justification of patients being involved in these studies, rather than getting proper palliative care. However, our study aims at exploring exactly these questions, as we learned from existing literature that palliative care is seldom offered to these people and that instead, experimental treatment then becomes almost a ‘default’ choice. With our study, we aimed to explore how this is organized in Belgium, as prior to our study, no knowledge of this topic was available. The current gap, therefore, was a lack of knowledge of how quality of life and palliative care are integrated in the trajectories of these patients, which our study aimed to address. We therefore feel that we cannot discuss the specifics of the needs of this patient group in the introduction, as our results and discussion address these needs.  However, we have addressed the justification as available in literature to involve patients in phase1 trials by expanding a section in the introduction as follows: “*Patients are motivated to participate in these trials out of hope for therapeutic benefit, with recent innovations in early-phase drug development having led to improved response rates and prolonged tumor response durations (3).”*  We have furthermore discussed the current scenario of palliative care in Belgium in general as follows *“Palliative care in Belgian hospitals can be provided either in a specialized way, provided in palliative care units by a team including specific palliative care experience, or in a generalist way, by multidisciplinary teams supporting patients not in a palliative care unit.”* | Lines 60-62, 91-93 |
| 3) The objectives 1 needs more clarity and objective two can be divided in two part. | We have rephrased objective 1 from “How is attention for quality of life integrated in the current care of cancer patients participating in phase I oncological trials?” to *“How are aspects of patients’ quality of life recognized, monitored, and supported within phase I oncological trials?”.*  We understand the reviewer questioning whether objective 2 should be divided into two parts, as it was previously phrased as follows: “How is palliative care currently addressed in phase I oncological trials, and how do patients and providers experience this?” We have split this into two sub-objectives, as we feel they still belong under the same umbrella objective, as follows:  *“2a. How is palliative care currently addressed in phase I oncological trials? 2b. What are patients’ attitudes towards palliative care provision?”* | Lines 109-113 |
| 4) Briefly mention how different perspectives will help will add to the strength of the study. This can come under the introduction. | We have clarified the purpose of our multi-perspective approach in the introduction section as follows:  “*As experiences and expectations around palliative care may differ markedly between patients, informal caregivers, and the various types of healthcare providers throughout patient’s care trajectory, exploring these multiple perspectives allows for a more comprehensive understanding of current practices and potential integration.”* | Lines 105-108 |
| 5) Qualitative research does not opt for quantification and thus do not include inter-rater reliability. This is usually reached through triangulation. | While we understand that many types of qualitative analysis methods, such as thematic analysis, indeed avoid quantification such as inter-rater reliability, our chosen analysis method, i.e. qualitative content analysis (QCA), prescribes the calculation of inter-rater reliability when multiple coders are involved, to assess the reliability of the coding scheme (rather than the reliability of the results which can indeed be reached through triangulation). See e.g.: Stevens P, editor. Qualitative data analysis : key approaches. London: SAGE; 2022. | N/A |
| 6) Review the analysis for more contents/themes.  -- The reviewer clarifies this further in the notes, commenting “can patients with strong will to live not be explained about PC since PC not necessarily mean end-of-life? does this indicate the held stigma among the staff?”. | We appreciate that the reviewer may feel that more themes may have been present in our data. However, when conducting our analysis, we reached data saturation as described in the methods section as follows*:*  *“The research team met regularly to discuss findings, adapt the topic guide, and assess data saturation, which was reached when no new key themes (e.g., new strategies or barriers) emerged.”*  We agree with the reviewer’s remark specifically about patients with strong will to live being explained about palliative care, and have already described this in the discussion (lines 536-542). | N/A |
| 7) Some of the claims as reported under the result are difficult to understand unless supported by sufficient participant narratives. Also the probes made to the patients seem directive which may have prevented a deeper perspectives from surfacing. | To enhance transparency, we added several additional illustrative quotes where the reviewer noted they would be useful.  Furthermore, we appreciate the reviewer’s remark on the phrasing of interview probes, which is likely based on the inclusion of the probe “And psychologically, have you experienced any issues because of the study?” in one of the illustrative quotes. We have adapted the presentation of this quote as to avoid the misunderstanding that this probing was particularly directive, as the context of this quote was that previously, physical issues had been discussed with the patient. In general, we wish to clarify that interviews were semi-structured and designed to allow participants to elaborate freely on their own experiences. | Lines 257-258, 270-271, 275-277 |
| 8) The results indicate towards many related issues, such as bureaucratic complication, knowledge and attitude towards palliative care which can be either mentioned in the discussion or as future implications  -- E.g., in the notes, the reviewer wonders whether the following may indicate a bureaucratic failure *“Patients indicated that once they started the oncological trajectory, their oncologist became their primary point of contact, leading them to less frequently visit the GP”.* | We agree with the reviewer that our results indicate issues regarding knowledge and attitudes towards palliative care, both on the level of patients and healthcare providers. We already discussed knowledge and attitude towards palliative care in the discussion (lines 536-546).  We believe our results do not allow us making inferences on bureaucratic barriers apart from those already mentioned in the discussion, specifically on fragmentation among healthcare providers (see lines 555-560). Based on our data, we do not see this as necessarily a bureaucratic failure, rather that patients simply prefer to stay with the oncologist instead of juggling multiple healthcare providers out of practical reasons. As such, we have not developed bureaucratic complications further in the discussion section, based on the findings of our study. | N/A |
