# Supplementary material for: Integration of palliative care into phase I oncology trials: A qualitative interview study with patients, informal caregivers, and healthcare providers
Source: PLoS One. 2026 May 5;21(5):e0347349. doi: 10.1371/journal.pone.0347349 (PMC13143114; doi:10.1371/journal.pone.0347349)
Supplement: Attachment — Submitted filename: PONE-D-25-38276_reviewer Early palliative Care.docx [file pone.0347349.s005.docx]

PLOS ONE

**Integration of palliative care into phase I oncology trials: A qualitative interview study with patients, informal caregivers, and healthcare providers**

--Manuscript Draft--

| **Manuscript Number:** | PONE-D-25-38276 |
| --- | --- |
| **Article Type:** | Research Article |
| **Full Title:** | Integration of palliative care into phase I oncology trials: A qualitative interview study with patients, informal caregivers, and healthcare providers |
| **Short Title:** | Integration of palliative care into phase I oncology trials |
| **Corresponding Author:** | Frederick Daenen  Vrije Universiteit Brussel Jette, BELGIUM |
| **Keywords:** | palliative care; phase I trial; quality of life |
| **Abstract:** | Background  Patients with advanced cancer participating in phase I clinical trials often face limited survival while experiencing significant symptom burden. Despite evidence supporting early palliative care integration alongside active cancer treatment to improve quality of life, the role of palliative care in phase I trials remains unclear.  Objective  To explore the perspectives of patients, informal caregivers, and healthcare providers on how quality of life and palliative care are currently addressed in phase I oncological trials, and to identify perceived benefits, barriers, and strategies for integrating palliative care.  Methods  We conducted a multi-perspective qualitative interview study across three Belgian university hospitals. The semi-structured interviews were analyzed using qualitative content analysis.  Results  Participants included sixteen patients, five informal caregivers, twelve phase I staff, six oncologists, five palliative care specialists, and four general practitioners. Patients generally reported positive experiences with trial participation, often viewing it as a final opportunity that provided hope and structure. However, quality of life support was inconsistently addressed and largely reactive. While patients reported feeling supported, non-trial providers and caregivers noted limited person-centered care. No systematic approach for introducing palliative care was in place. Palliative care was rarely discussed, hindered by misconceptions such as equating palliative care with terminal care, reluctance from patients and clinicians, and lack of communication between providers. Participants suggested the introduction of routine yet flexible palliative care conversations, as well as improved communication between providers, as strategies towards integration.  Conclusion  Despite recognized care needs, palliative care is not systematically integrated in phase I oncological trials. Quality of life remains a secondary concern. Integrating palliative care in a structured yet flexible manner could support more holistic, patient-centered care. These findings underscore the need to normalize palliative care as a complementary component of phase I oncological trials. |
| **Order of Authors:** | Frederick Daenen |
|  | Anne van Driessche |

|  | Anne-Lore Scherrens |
| --- | --- |
|  | Annelies Janssens |
|  | Sylvie Rottey |
|  | Lore Decoster |
|  | Peter A. J. Stevens |
|  | Koen Pardon |
|  | Aline De Vleminck |
|  | Kim Beernaert |
| **Additional Information:** |  |
| **Question** | **Response** |
| **Financial Disclosure**  Enter a financial disclosure statement that describes the sources of funding for the work included in this submission. Review the [submission guidelines](http://journals.plos.org/plosone/s/submission-guidelines#loc-financial-disclosure-statement) for detailed requirements. View published research articles from [*PLOS ONE*](https://journals.plos.org/plosone/) for specific examples.  This statement is required for submission and **will appear in the published article** if the submission is accepted. Please make sure it is accurate. | Yes |
| **Funded studies**  Enter a statement with the following details:   - Initials of the authors who received each award - Grant numbers awarded to each author - The full name of each funder - URL of each funder website - Did the sponsors or funders play any role in the study design, data collection and analysis, decision to publish, or preparation of the manuscript? |  |
| Did you receive funding for this work? |  |
| Please add funding details.  as follow-up to "**Financial Disclosure**  Enter a financial disclosure statement that describes the sources of funding for the work included in this submission. Review the [submission guidelines](http://journals.plos.org/plosone/s/submission-guidelines#loc-financial-disclosure-statement) for detailed | Project funded by Kom op tegen Kanker (Stand up to Cancer), the Flemish cancer society (13719) (URL: [https://www.komoptegenkanker.be/).](http://www.komoptegenkanker.be/)) This grant was awarded to author KB. The funder had no role in study design, data collection and analysis, decision to publish, or preparation of the manuscript. |

| requirements. View published research articles from [*PLOS ONE*](https://journals.plos.org/plosone/) for specific examples.  This statement is required for submission and **will appear in the published article** if the submission is accepted. Please make sure it is accurate. |  |
| --- | --- |
| **Funded studies**  Enter a statement with the following details:   - Initials of the authors who received each award - Grant numbers awarded to each author - The full name of each funder - URL of each funder website - Did the sponsors or funders play any role in the study design, data collection and analysis, decision to publish, or preparation of the manuscript? |  |
| Did you receive funding for this work?" |  |
| Please select the country of your main research funder (please select carefully as in some cases this is used in fee calculation).  as follow-up to "**Financial Disclosure**  Enter a financial disclosure statement that describes the sources of funding for the work included in this submission. Review the [submission guidelines](http://journals.plos.org/plosone/s/submission-guidelines#loc-financial-disclosure-statement) for detailed requirements. View published research articles from [*PLOS ONE*](https://journals.plos.org/plosone/) for specific examples.  This statement is required for submission and **will appear in the published article** if the submission is accepted. Please make sure it is accurate. | BELGIUM - BE |

| **Funded studies**  Enter a statement with the following details:   - Initials of the authors who received each award - Grant numbers awarded to each author - The full name of each funder - URL of each funder website - Did the sponsors or funders play any role in the study design, data collection and analysis, decision to publish, or preparation of the manuscript? |  |
| --- | --- |
| Did you receive funding for this work?" |  |
| **Competing Interests**  Use the instructions below to enter a competing interest statement for this submission. On behalf of all authors, disclose any [competing interests](http://journals.plos.org/plosone/s/competing-interests) that could be perceived to bias this work—acknowledging all financial support and any other relevant financial or non- financial competing interests.  This statement is required for submission and **will appear in the published article** if the submission is accepted. Please make sure it is accurate and that any funding sources listed in your Funding Information later in the submission form are also declared in your Financial Disclosure statement.  View published research articles from  [*PLOS ONE*](http://journals.plos.org/plosone/) for specific examples. | The authors have declared that no competing interests exist. |

| **NO authors have competing interests**  Enter: *The authors have declared that no competing interests exist.*  **Authors with competing interests**  Enter competing interest details beginning with this statement:  *I have read the journal's policy and the authors of this manuscript have the following competing interests: [insert competing interests here]* |  |
| --- | --- |
| * typeset |  |
| **Ethics Statement**  Enter an ethics statement for this submission. This statement is required if the study involved:   - Human participants - Human specimens or tissue - Vertebrate animals or cephalopods - Vertebrate embryos or tissues - Field research   Write "N/A" if the submission does not require an ethics statement.  General guidance is provided below. Consult the [submission guidelines](http://journals.plos.org/plosone/s/submission-guidelines#loc-guidelines-for-specific-study-types) for detailed instructions. **Make sure that all information entered here is included in the Methods section of the manuscript.** | This study was approved by the central ethics committees of Ghent University Hospital (6702022000253) and UZ Brussel (1432023000251) and by the local committee of Antwerp University Hospital.  All participants received study information and gave written informed consent. |

| **Format for specific study types**  **Human Subject Research (involving human participants and/or tissue)**   - Give the name of the institutional review board or ethics committee that approved the study - Include the approval number and/or a statement indicating approval of this research - Indicate the form of consent obtained (written/oral) or the reason that consent was not obtained (e.g. the data were analyzed anonymously)   **Animal Research (involving vertebrate animals, embryos or tissues)**   - Provide the name of the Institutional Animal Care and Use Committee (IACUC) or other relevant ethics board that reviewed the study protocol, and indicate whether they approved this research or granted a formal waiver of ethical approval - Include an approval number if one was obtained - If the study involved *non-human primates*, add *additional details* about animal welfare and steps taken to ameliorate suffering - If anesthesia, euthanasia, or any kind of animal sacrifice is part of the study, include briefly which substances and/or methods were applied   **Field Research**  Include the following details if this study involves the collection of plant, animal, or other materials from a natural setting:   - Field permit number - Name of the institution or relevant body that granted permission |  |
| --- | --- |
|  |  |
| **Data Availability**  Authors are required to make all data underlying the findings described fully available, without restriction, and from the time of publication. PLOS allows rare exceptions to address legal and ethical concerns. See the [PLOS Data Policy](http://journals.plos.org/plosone/s/data-availability) and [FAQ](http://journals.plos.org/plosone/s/data-availability#loc-faqs-for-data-policy) for detailed information. | No - some restrictions will apply |

| A Data Availability Statement describing where the data can be found is required at submission. Your answers to this question constitute the Data Availability Statement and **will be published in the article**, if accepted. |  |
| --- | --- |
| **Important:** Stating ‘data available on request from the author’ is not sufficient. If your data are only available upon request, select ‘No’ for the first question and explain your exceptional situation in the text box. |  |
| Do the authors confirm that all data underlying the findings described in their manuscript are fully available without restriction? |  |
| **Describe where the data may be found in full sentences. If you are copying our sample text, replace any instances of XXX with the appropriate details.**   - If the data are **held or will be held in a public repository**, include URLs, accession numbers or DOIs. If this information will only be available after acceptance, indicate this by ticking the box below. For example: *All XXX files are available from the XXX database (accession number(s) XXX, XXX.*). - If the data are all contained **within the manuscript and/or Supporting Information files**, enter the following: *All relevant data are within the manuscript and its Supporting Information files.* - If neither of these applies but you are able to provide **details of access elsewhere**, with or without limitations, please do so. For example:   *Data cannot be shared publicly because of [XXX]. Data are available from the XXX Institutional Data Access / Ethics Committee (contact via XXX) for researchers who meet the criteria for access to confidential data.*  *The data underlying the results presented in the study are available from (include the name of the third party* | As the data concern interview transcripts with participants, including patients, family caregivers, and healthcare professionals, and as they as such contain personal data, they cannot be shared publicly. Requests for pseudonymized versions of the data may be addressed to the first author. Every request will be evaluated on an individual basis and the ethics committee of the Vrije Universiteit Brussels will be contacted for approval before any sharing of participant-level data. |

| *and contact information or URL).*   - This text is appropriate if the data are owned by a third party and authors do not have permission to share the data.   * typeset |  |
| --- | --- |
| Additional data availability information: |  |

Manuscript	[Click here to access/download;Manuscript;Manuscript.docx](https://www2.cloud.editorialmanager.com/pone/download.aspx?id=41284450&guid=5b549138-f9a3-4b33-b33d-55b84164ee8e&scheme=1)[<https://www2.cloud.editorialmanager.com/pone/download.aspx?id=41284450&guid=5b549138-f9a3-4b33-b33d-55b84164ee8e&scheme=1>](https://www2.cloud.editorialmanager.com/pone/download.aspx?id=41284450&guid=5b549138-f9a3-4b33-b33d-55b84164ee8e&scheme=1)

1 **Title: Integration of palliative care into phase I oncology trials: A qualitative interview**
2 **study with patients, informal caregivers, and healthcare providers**

# 3

4	Short title: Integration of palliative care into phase I oncology trials

# 5

6 Frederick Daenen 1,2,*, Anne van Driessche 1,2, Anne-Lore Scherrens 1,3, Annelies
7 Janssens 4, Sylvie Rottey 5, Lore Decoster 6, Peter A. J. Stevens 7, Koen Pardon 1,2, Aline
8 De Vleminck 1,2, Kim Beernaert 1,3

# 9

10 1 End-of-life Care Research Group, Vrije Universiteit Brussel (VUB) & Ghent University,
11 Brussels, Belgium
12 2 Department of Family Medicine and Chronic Care, Vrije Universiteit Brussel (VUB),
13 Brussels, Belgium
14 3 Department of Public Health and Primary Care, Ghent University, Gent, Belgium
15 4 Department of Pulmonology and Thoracic Oncology, Antwerp University Hospital, Antwerp,
16 Belgium
17 5 Department of Medical Oncology, Ghent University Hospital, Ghent, Belgium
18 6 Department of Medical Oncology, Oncologisch Centrum, Universitair Ziekenhuis Brussel,
19 Vrije Universiteit Brussel, Brussels, Belgium
20 7 Department of Sociology, Faculty of Political and Social Sciences, Ghent University,
21 Ghent, Belgium
22 * Corresponding author; E-mail: [Frederick.daenen@vub.be](mailto:Frederick.daenen@vub.be)
23 **Abstract**

# 24

25 Background: Patients with advanced cancer participating in phase I clinical trials often face
26 limited survival while experiencing significant symptom burden. Despite evidence supporting
27 early palliative care integration alongside active cancer treatment to improve quality of life,
28 the role of palliative care in phase I trials remains unclear.

# 29

30 Objective: To explore the perspectives of patients, informal caregivers, and healthcare
31 providers on how quality of life and palliative care are currently addressed in phase I
32 oncological trials, and to identify perceived benefits, barriers, and strategies for integrating
33 palliative care.

# 34

35 Methods: We conducted a multi-perspective qualitative interview study across three Belgian
36 university hospitals. The semi-structured interviews were analyzed using qualitative content
37 analysis.

# 38

39 Results: Participants included sixteen patients, five informal caregivers, twelve phase I staff,
40 six oncologists, five palliative care specialists, and four general practitioners. Patients
41 generally reported positive experiences with trial participation, often viewing it as a final
42 opportunity that provided hope and structure. However, quality of life support was
43 inconsistently addressed and largely reactive. While patients reported feeling supported,
44 non-trial providers and caregivers noted limited person-centered care. No systematic
45 approach for introducing palliative care was in place. Palliative care was rarely discussed,
46 hindered by misconceptions such as equating palliative care with terminal care, reluctance
47 from patients and clinicians, and lack of communication between providers. Participants
48 suggested the introduction of routine yet flexible palliative care conversations, as well as
49 improved communication between providers, as strategies towards integration.

# 50

51 Conclusion: Despite recognized care needs, palliative care is not systematically integrated in
52 phase I oncological trials. Quality of life remains a secondary concern. Integrating palliative
53 care in a structured yet flexible manner could support more holistic, patient-centered care.
54 These findings underscore the need to normalize palliative care as a complementary
55 component of phase I oncological trials.
56 **Introduction**

# 57

58 Patients with advanced cancer who have exhausted standard treatment options can be
59 offered participation in phase I clinical trials (1). These trials primarily focus on evaluating the
60 toxicity and appropriate dosing of novel therapies, unlike phase II and phase III trials which
61 assess therapeutic efficacy (2). Although recent innovations in early-phase drug
62 development have led to improved response rates and prolonged tumor response
63 durations(3), most phase I trial participants face a limited survival outlook, with the majority
64 passing away within a year (4,5). Importantly, while eligibility criteria for phase I trials
65 generally require patients to have a relatively good performance status (ECOG 0–1) (6),
66 retrospective studies show that these patients experience a high symptom burden,
67 comparable to or even greater than that of patients who are not enrolled in clinical trials (6–
68 8).

# 69

70 Despite patients’ significant care needs, the primary focus in phase I studies remains on
71 experimental treatment (6). Patients are often confronted with a choice between pursuing
72 experimental therapy or transitioning fully to comfort-oriented, palliative care (9,10). In this
73 context, healthcare providers play a crucial role not only in screening and enrolling eligible
74 patients, but also in providing clear information about the trial’s aims, limitations, and
75 potentially also about palliative care, balancing transparency with protection from potential
76 overtreatment (11).

# 77

78 Palliative care is a holistic approach that addresses physical, psychological, social, and
79 spiritual needs, and may be provided by palliative care specialists or integrated through
80 basic palliative care principles by other healthcare providers (12). This may include a holistic
81 focus on the quality of life of patients in the broadest sense, as well as more specific actions
82 such as the discussion of advance care planning and palliative care services. Early
83 integration of palliative care alongside active cancer treatment has been shown to improve
84 quality of life for patients and their families (13–16). For patients who are no longer
85 candidates for curative treatment, early discussions about goals of care, advance care
86 planning, and the potential initiation of palliative services are critical (13,17,18). In Belgium,
87 phase I trials are primarily conducted at large academic centers, with other hospitals
88 referring patients to these centers. Coordination of care can become more complex when
89 patients transition between centers or departments, making attention to quality of life,
90 advance care planning, and palliative care even more essential.

# 91

92 At present, little is known about whether palliative care is integrated in phase I trials: whether
93 and how palliative care is discussed with this patient population, when it is initiated, and how
94 patients and families experience their quality of life during participation in experimental
95 studies. Given the significant care needs and limited survival outlook of trial participants,
96 early integration of palliative care could offer substantial benefits.

# 97

98 **Objectives**
99 The goal of this project is to gain insights into the following three research questions, as
100 perceived by trial participants with cancer, informal caregivers, and various types of
101 healthcare providers (phase I staff, referring oncologists, general practitioners, and palliative
102 care specialists).

How is attention for quality of life integrated in the current care of cancer patients participating in phase I oncological trials?

| 103 | 1. |
| --- | --- |
| 104 |  |
| 105 | 2. |
| 106 |  |
| 107 | 3. |
| 108 |  |
| 109 |  |
| 110 |  |

How is palliative care currently addressed in phase I oncological trials, and how do patients and providers experience this?

What are the perceived benefits, barriers, and strategies to integrating palliative care into phase I oncological trials?

**Method**

111	**Study design**

# 112

113 This study employed a qualitative research design using semi-structured face-to-face
114 interviews. A qualitative approach was chosen as it allows for the in-depth studying of
115 unexplored topics, with semi-structured interviews allowing flexibility for the uncovering of
116 potentially unforeseen topics related to the research questions. We applied a multi-
117 perspective essentialist approach, prioritizing a broader data collection of multiple types of
118 participants over a more idiosyncratic hermeneutical interpretative approach (19). This study
119 was conducted and reported following the Consolidated Criteria for Reporting Qualitative
120 Research (COREQ) guidelines (S1 COREQ cheklist) (20).

# 121

122	**Population and setting**

# 123

124 To comprehensively explore perspectives on integrating palliative care into phase I clinical
125 trials, we included cancer patients, informal caregivers, and healthcare providers. The latter
126 group comprised phase I staff (physicians, nurses, study coordinators), referring oncologists,
127 GPs, and palliative care specialists (i.e., members of the hospital palliative support team).
128 We targeted at least four participants per category and at least five staff members per site.
129 The study was conducted at three Belgian university hospitals: Ghent University Hospital
130 and Antwerp University Hospital (both with dedicated oncological trial units) and UZ Brussel
131 (no dedicated unit but referring patients to such units and hosting small-scale trials). This
132 study was approved by the central ethics committees of Ghent University Hospital
133 (6702022000253) and UZ Brussel (1432023000251) and by the local committee of Antwerp
134 University Hospital.

# 135

136 Inclusion criteria required participants to be Dutch-speaking and aged 18 or older. Patients
137 had to be enrolled in any phase I oncological trial; informal caregivers were those caring for
138 a patient in such a trial. Healthcare providers needed at least one year of experience in their
139 current role. Non-phase I staff needed experience with phase I patients, either through
140 referrals or managing patients in trials. Providers were not necessarily treating the patients
141 interviewed.

# 142

143	**Recruitment**

# 144

145 Participants were recruited using convenience and snowball sampling. Recruitment started
146 at Ghent University Hospital on September 1^st^ 2022, with Antwerp University Hospital and
147 UZ Brussel added as study sites on March 1^st^ 2024. Recruitment ended at all sites on July
148 2^nd^ 2024. Patients and informal caregivers were approached face-to-face by a study
149 coordinator based on expected burden, with interested individuals referred to the research
150 team who contacted them via telephone. Non-participation was mainly due to health issues
151 or reluctance to engage in further research. Healthcare professionals were recruited via
152 networks and snowballing. All participants received study information and gave written
153 informed consent.

# 154

155	**Data collection**

# 156

157 Interviews took place between September 19^th^ 2022 and July 2^nd^ 2024 at locations chosen
158 by participants. Patients and caregivers were interviewed during or after hospital visits, and
159 healthcare providers during working hours. In two cases, patients and informal caregivers
160 were interviewed together. A stakeholder-informed topic guide was tailored for each group,
161 covering expectations toward the trial, quality of life, advance care planning, and palliative
162 care (S2 Topic guides). To minimize bias, participants first shared their understanding of
163 palliative care before being provided with a broad, holistic definition. Sociodemographic data
164 were collected via brief questionnaires.

# 165

166 Interviews were conducted in Dutch by two postdoctoral researchers (FD, AVD), one PhD
167 student in psychology, and two trained master’s students in healthcare. Interviewers took
168 post-interview field notes. All interviews were audio-recorded, transcribed verbatim, and
169 ranged from 28–117 minutes (patients/caregivers) and 32–95 minutes (healthcare
170 providers). No transcripts had to be returned to participants. The research team met
171 regularly to discuss findings, adapt the topic guide, and assess data saturation, which was
172 reached when no new key themes (e.g., new strategies or barriers) emerged.

# 173

174	**Data analysis**

# 175

176 Data were analyzed using qualitative content analysis with NVivo 14 (21). Qualitative content
177 analysis was selected for its suitability in systematically identifying categories within large
178 datasets and supporting descriptive exploration in understudied areas (19,22,23), combining
179 inductive and deductive approaches as described below (24,25). As our semi-structured
180 interviews do not allow direct comparison of category prevalence, we focus on a narrative
181 presentation of findings without quantification (19).

# 182

183 The coding process involved two coders (FD, AVD) first familiarizing themselves with the
184 data, then independently performing ‘open coding’ on a subset of five interviews, selected
185 according to the diversity principle, i.e., having at least one interview per participant category
186 and hospital. Open coding refers to inductively attributing codes to small, meaningful parts of
187 the transcripts relating to our research questions, with codes describing the essence of the
188 coded text while staying close to the raw data (24,26). The coders then collaboratively and
189 iteratively developed a coding tree, grouping inductively applied open codes into
190 (sub)categories, deductively informed by categories of interest for the research questions: 1)
191 quality of life, 2) current palliative care, and 3) potential integration of palliative care, broadly
192 including palliative care communication, advance care planning, and focusing on quality of
193 life. In a next step, the coders developed a hierarchical coding scheme based on the coding
194 tree, including definitions and applicability rules for all (sub)categories. After the development
195 of the initial coding scheme, trial coding was conducted by both coders independently
196 applying the coding scheme to another subset of selected interviews until a strong interrater
197 reliability of .70 was reached, assessed with Cohen’s kappa, iteratively refining the coding
198 scheme after each round of trial coding. This threshold was reached after the independent
199 coding of four interviews, resulting in a finalized coding scheme (see Table 1) (27,28). The
200 finalized coding scheme was then applied to all transcripts, with coders dividing the
201 remaining transcripts between them. After coding, key findings within each subcategory were
202 synthesized per participant category using a table with each cell representing the main
203 findings per theme (columns) and interview (rows). Representative quotes selected to
204 illustrate the key findings in each subcategory. To enhance the validity of the findings, we
205 applied member checking, sharing a synthesis of the results with a subset of 8 participants,
206 including representatives from each participant type, to verify the interpretation and add
207 further nuance where needed. Interviewed participants supported the shared findings – small
208 nuances to the findings emerging from the member checking interviews are integrated in the
209 results section below.

# 210

211

**Table 1. Coding scheme.**

| **Category** | **Description** | **Subcategories & description** | **Applicability** | **Example codes** |
| --- | --- | --- | --- | --- |
| Quality of life in phase I studies | This category includes subcategories related to the quality of life of participants in Phase I studies and their  relatives, as experienced throughout the Phase I trajectory. | Subcategories are classified based on:   1. The reasons patients & relatives participate in the study (what do they hope to achieve?); 2. The experienced quality of life, in the broadest sense, of the patient and their relative during the study; 3. The extent to which study personnel do or do not take their quality of life into account (separately from the concept of palliative care). | Statements are coded regardless of whether they come from the patient, relative, or healthcare provider, but they must  concern the quality of life of the patient or their relative. | Participating due to a final life wish  Being able to live as normally as possible  Feeling lonely in the hospital room |
| Palliative care in the context of phase I studies | This category includes all subcategories related to palliative care in the phase I trajectory. Palliative care is considered holistically but must be explicitly understood or discussed in terms of palliative care – general quality-of-life considerations are not coded under this category. | Subcategories are classified based on:   1. Patients’ & relatives’ attitudes towards palliative care, specifically also regarding advance care planning; 2. How palliative care is or is not discussed with the patient by healthcare providers, and in what manner; 3. Communication about palliative care for the patient between different healthcare providers. | Statements are coded as long as they explicitly relate to the concept of palliative care within the study trajectory, starting from the referral. | Discussing palliative care is for later  Palliative care is not discussed with the patient  Palliative care is not mentioned at the time of referral |
| Integration | This category includes all reflections from patients,  relatives, or healthcare providers on the potential integration of palliative care within the study trajectory. | Subcategories are classified based on the characteristics of the integration:   1. The benefits of such integration; 2. The concrete approach to such integration, including role distribution; 3. The barriers to such integration. | Disadvantages are placed under barriers. Barriers are also interpreted as reasons why palliative care is not yet provided. However, the absence of palliative care is coded in the above category (discussion of palliative care). | Patients need to know the reality of their situation  The oncologist should mention palliative care at the time of  referral  It is too early for this population |

212	**Results**

# 213

214 A total of 48 participants were interviewed: sixteen patients, five informal caregivers, twelve
215 phase I staff, six oncologists, five palliative care specialists, and four GPs. Participants
216 characteristics are displayed in Table 2. Cancer types and stages varied across participating
217 patients. Phase I staff members held a diverse range of roles, including four physician-
218 investigators, two heads of department, three nurses, and three study coordinators.

219

220

221

**Table 2. Participant characteristics.**

|  |  | **Patients** | **Informal caregivers** | **Phase I staff** | **Oncologists** | **Palliative care specialists** | **General practitioners** |
| --- | --- | --- | --- | --- | --- | --- | --- |
| **Total N** |  | **16** | **5** | **12^a^** | **6** | **5** | **4** |
| Sex | Female | 4 (25%) | 2 (40%) | 8 (67%) | 6 (100%) | 4 (80%) | 3 (75%) |
| Age | 18-30 | 0 (0%) | 1 (20%) | 2 (17%) | 0 (0%) | 0 (0%) | 0 (0%) |
|  | 31-50 | 1 (6%) | 0 (0%) | 8 (67%) | 5 (83%) | 1 (20%) | 1 (25%) |
|  | 51-70 | 10 (63%) | 3 (60%) | 2 (17%) | 1 (17%) | 4 (80%) | 3 (75%) |
|  | 70+ | 4 (25%) | 1 (20%) | 0 (0%) | 0 (0%) | 0 (0%) | 0 (0%) |
|  | missing | 1 (6%) | 0 (0%) | 0 (0%) | 0 (0%) | 0 (0%) | 0 (0%) |
| Location | UZ Brussel | 0 (0%) | 0 (0%) | 2 (17 %) | 2 (33%) | 0 (0%) | 0 (0%) |
|  | Ghent University Hospital | 12 (75%) | 4 (80%) | 6 (50%) | 2 (33%) | 2 (40%) | 0 (0%) |
|  | Antwerp University Hospital | 4 (25%) | 1 (20%) | 4 (33%) | 2 (33%) | 2 (40%) | 0 (0%) |
|  | Outside hospitals | 0 (0%) | 0 (0%) | 0 (0%) | 0 (0%) | 1 (20%) | 4 (100%) |
| Education | Higher education | 10 (63%) | 3 (60%) |  |  |  |  |
|  | High school | 4 (25%) | 1 (20%) |  |  |  |  |
|  | None | 1 (6%) | 0 (0%) |  |  |  |  |
|  | missing | 1 (6%) | 1 (20%) |  |  |  |  |
| Marital status | Married | 14 (88%) | 5 (100%) |  |  |  |  |
|  | Divorced | 1 (6%) | 0 (0%) |  |  |  |  |
|  | Widowed | 1 (6%) | 0 (0%) |  |  |  |  |
| Religion | Catholic | 6 (38%) | 1 (20%) |  |  |  |  |
|  | Atheist | 7 (44%) | 2 (40%) |  |  |  |  |
|  | Other | 3 (19%) | 1 (20%) |  |  |  |  |
|  | missing | 0 (0%) | 1 (20%) |  |  |  |  |
| Years of experience in current role (M, SD)b | |  |  | 8,6 (2,3) | 11,3 (2,8) | 15,3 (3,8) | 23,3 (5,1) |

a. Consisting of: 6 physicians, 3 study coordinators, 3 nurses.
b. Means (M) and standard deviations (SD) are displayed for years of experience of healthcare professionals in their current role.
222 The findings are grouped according to the three research aims, each including three
223 subcategories: first, quality of life, including reasons for participation, experienced quality of
224 life, and staff efforts to support quality of life; second, palliative care, including patient
225 attitudes, current communication with patients, and current communication between
226 healthcare providers; third, the potential integration of palliative care in the future, including
227 benefits, barriers, and strategies. For each subcategory, we narrate the key findings and
228 include supporting representative quotes. A summary of the (sub)categories can be found in
229 Figure 1.

# 230

231	**Figure 1. Summary of (sub)categories and findings.**

232	(see figure uploaded separately)

# 233

234	**Quality of life in phase I clinical trials**

# 235

236	Quality of life as a motivation for participation

# 237

238 Participants reported multiple reasons for enrolling in a phase I clinical trial, many of which
239 reflected their hopes of preserving or improving quality of life. Hope for therapeutic benefit
240 was the most typical factor, particularly among younger patients – even though they
241 acknowledged that physicians had been transparent about the lack of guaranteed
242 improvement. This is illustrated by the following quotes from a patient (P) and their informal
243 caregiver (IC):

# 244

245 *“(P) I hope – expectations are something else – I hope it works, yes.*
246 *(IC) While they haven’t... given her any reason for hope, that’s not it. That has been made*
247 *clear, it’s an experiment. So, it doesn’t cure, but it can of course prolong and keep (her) in*
248 *good condition.*
249 *(P) So nothing about recovery or so, I know that’s not possible.” (Patient & informal*
250 *caregiver)*

# 251

252 Many participants saw trial participation as their last available option for treatment – a choice
253 that could stabilize their condition and provide structured follow-up, reducing fears of being
254 left without support and maintaining quality of life in the face of uncertainty. Some also cited
255 non-personal motivations, such as altruism and contributing to science, while others framed
256 their decision relationally, seeking to extend life for loved ones or reach important
257 milestones. Healthcare providers largely echoed these motivations, noting that patients who
258 sign up often have a strong wish for continued treatment. However, some providers pointed
259 out that patients may feel pressured to participate when suggested by their oncologist.

# 260

261	Experienced quality of life and well-being

# 262

263 Patients overwhelmingly described their quality of life and well-being during the trial as
264 positive, specifically due to symptom improvement and to the psychological boost and hope
265 provided by having this, as they often perceived it, ‘final chance’. This is illustrated
266 particularly well by the following quote where a patient notes that participation grants
267 perspective that otherwise would not have been there: *“(And psychologically, have you*
268 *experienced any issues because of the study?) Because of the study? Certainly not,*
269 *because the study gives hope, of course, so it’s the opposite. If you have nothing left, I think*
270 *that’s more difficult.” (Patient)*

# 271

272 Patients often expressed great gratitude for being afforded the opportunity to participate in
273 the trial. They also noted feeling secure due to the 24/7 availability of phase I staff, signifying
274 stronger monitoring than during standard oncological treatment.

# 275

276 Nevertheless, challenges were also reported. Besides the potential side effects, which varied
277 greatly across treatments, uncertainty regarding treatment effectiveness and long-term
278 outcomes was a source of distress for some, with some patients describing a sense of being
279 ‘guinea pigs’: *“I am a guinea pig. I’m not really a patient here, if you know what I mean –*
280 *they’re observing what the drug does to me in a strictly scientific way.” (Patient)*

# 281

282 Additionally, the time demands of the trial, including frequent hospital visits and extensive
283 testing, were perceived as burdensome by all types of interviewed participants, although less
284 so by the patients than the other interviewees.

# 285

286	Staff efforts to support quality of life

# 287

288 Phase I staff expressed routinely verbally inquiring about quality of life, which patients
289 echoed, who generally indicated feeling heard. Phase I staff noted that they would refer
290 patients to psychologists or dieticians, although only reactively upon noticing a clear need,
291 rather than proactively. However, informal caregivers and non-trial healthcare providers were
292 less positive, highlighting a gap in psychosocial support. Notably, while phase I staff
293 indicated that they understood quality of life to be highly subjective based on each patient
294 and what they deemed to important for their own lives, this was not reflected by the
295 perception of informal caregivers and non-trial providers, who indicated that inquiring into
296 patients’ quality of life remains surface-level and that how the patient is ‘really’ doing is often
297 not addressed:

# 298

299 *“It’s never about quality of life. It’s always about objective things [medical results].” (GP)*
300 *“What the patient reports, often those are questionnaires or an interview with a psychologist,*
301 *but that’s more in phase II or phase III studies. What the patient thinks is less considered in*
302 *phase I.” (Oncologist)*

# 303

304 While phase I staff indicated that patients sometimes complete quality of life questionnaires,
305 this was inconsistent across studies. When such questionnaires were administered, staff
306 members admitted that the results were typically not available to them, only being collected
307 for study purposes and not being acted upon. While personal goals and wishes of patients
308 were not always explicitly discussed (e.g., preferences for timing of treatments), phase I staff
309 did report making efforts to balance patients’ personal preferences with trial protocol
310 requirements, although this also highlights the concessions that patients may have to make
311 to remain with the boundaries of the study protocols:

# 312

313	*“That’s not always in our hands, we’re somewhat bound to the protocols that must be*

314	*followed. But of course, the patient’s wish is always the most important. We certainly take*

315	*that into account. If they report something like, look, that really wouldn’t work, then we try to*

316	*adjust.” (Phase I study coordinator)*

# 317

318	**Current palliative care in phase I clinical trials**

# 319

320	Patient attitudes towards palliative care

# 321

322 Patients mostly held negative attitudes and resistance towards the concept of palliative care,
323 associating it with terminal care. Many did not consider themselves as ‘palliative’ – nor
324 terminal – and perceived conversations about palliative care as premature or unnecessary,
325 as illustrated by the following quotes:

# 326

327 *“I’ve never been in that mindset of ‘it won’t be long – I have two months to live.’ I’ve never*
328 *been there. It’s also never been said to me. If they told me tomorrow, ‘sorry, you have two*
329 *months left.’ Only then would I consider myself truly palliative and prepare my mind for*
330 *death. But I’m not occupied with that yet. That’s not the case now, not yet.” (Patient)*
331 *“(And has the word palliative care ever come up? Has the doctor or a nurse ever mentioned*
332 *it?) No. But I think they also know not to bring it up with me.” (Patient)*

# 333

334 Most patients interviewed appeared unaware of the broader scope of palliative care and
335 benefits it could offer; those who were aware, attributed their knowledge to doing their own
336 research, rather than being informed by healthcare providers.

# 337

338	Communication about palliative care with patients

# 339

340 While phase I staff acknowledged the importance of – and their responsibility in – discussing
341 palliative care, there was no systematic integration of palliative care conversations within the
342 phase I trial setting. Conversations about palliative care were rarely held in the context of the
343 phase I trial setting, either remaining unaddressed or being postponed until patients’
344 condition worsened significantly, in which case staff would refer to palliative care specialists
345 who would thus only be involved reactively in their care trajectories. They also noted that the
346 study population has a particularly very strong will to live, indicating to them that discussing
347 palliative care is likely not welcomed. Some phase I staff felt that the context of a phase I
348 trial did not easily allow for a focus on palliative care:

# 349

350 *“Yes, I think, time-wise. We have to discuss a lot of practical things, a lot about the study.*
351 *Hm, and it also falls somewhere within that… but it’s also about balancing giving hope while*
352 *we are focused on the side effects, right? Yes, sometimes that just falls by the wayside, I*
353 *must say.” (Phase I physician)*

# 354

355 Taking into account this difficult context, phase I staff often indicated that the task of bringing
356 up palliative care may be more suited for the referring oncologist, as patients typically do not
357 remain sufficiently long in the trial to build up a strong connection:

# 358

359 *“In phase I, [palliative care] is not immediately actively started, I think, or only rarely.*
360 *Because those patients are usually referred by the treating oncologist, since you’re*
361 *somewhat the interim oncologist. They’ve usually been in a palliative trajectory for quite*
362 *some time. So I’m not with the patient... That might be something I pay too little attention to.*
363 *But with the idea that, yes, that might already have been started by the treating oncologist.”*
364 *(Phase I physician)*

# 365

366 However, referring oncologists noted that referral to a phase I trial was a barrier for them to
367 start such conversations, as it seemed too much of a mixed message to the patient to them.
368 Importantly, some healthcare providers, whether phase I staff or referring oncologists,
369 expressed that they did bring up palliative care with their patients, but attributed this to a
370 personal conviction of its benefits rather than a systematic approach. When palliative care
371 was addressed, many healthcare providers avoided using the term ‘palliative’, instead opting
372 for more neutral language such as comfort care. However, some providers, mainly palliative
373 care specialists, expressed the importance of using the term ‘palliative’ as to avoid the
374 patient not understanding the severity of the situation, while offering an explanation to the
375 patient of what the term precisely entails.

# 376

377	Communication about palliative care between healthcare providers

# 378

379 Information exchange about palliative care between healthcare providers was inconsistent.
380 While the referring oncologist is asked to provide a large amount of information to the phase
381 I unit upon their referral, details on palliative care status, needs, or conversations are neither
382 asked, nor systematically shared. While some oncologists chose to provide this information
383 organically, this was limited and based on personal conviction rather than standardized
384 practice. As such, phase I staff also often were unaware of whether palliative care
385 conversations had been held with the patients prior to their enrollment in the trial trajectory,
386 or even whether palliative care support at home had been setup:

# 387

388 *“(Are you aware of whether, when people come here, [palliative care] has already been*
389 *discussed?) I don’t always know that. Usually or often it is, but not always, or they haven’t*
390 *remembered it. But I don’t know in advance, systematically, what they already know about it*
391 *and what they don’t.” (Phase I physician)*

# 392

393 During the phase I trial, communication between the phase I unit and referring oncologists or
394 GPs was notably limited. Although a system of notification letters is used by the phase I staff
395 to keep the referring oncologist or GP up to date, the latter groups lamented the lack of
396 useful information shared through these letters, often focusing on highly specific medical
397 details and not including any information on palliative care. Referring oncologists noted that
398 they often ‘lose’ a patient once they start participation in a phase I trial, even though they feel
399 they might play a role in further follow-up, including palliative care. GPs specifically felt that a
400 lack of communication resulted in them not being able to take up palliative care
401 conversations with the patient, which they deemed to be a core task given their often
402 longstanding relationship with the patient. Instead, they typically only see the patient once
403 they are already dismissed from the trial, often much later than they had preferred to still
404 play a meaningful role in their care. Patients indicated that once they started the oncological
405 trajectory, their oncologist became their primary point of contact, leading them to less
406 frequently visit the GP.

# 407

408	**Potential integration of palliative care in phase I clinical trials**

# 409

410	Perceived benefits of integration

# 411

412 Patients typically indicated having no need for palliative care – at least at the stage where
413 they were at. Informal caregivers were generally more open to the idea of standardized
414 integration of palliative care in the phase I trajectories, noting that they themselves also had
415 information and psychosocial support needs resulting from the patient’s illness. They also
416 expressed that some patients may not be sufficiently assertive to demand forms of palliative
417 care if it is not actively offered to them. Healthcare providers identified several potential
418 benefits of integrating palliative care into phase I trials, mainly centered around providing
419 less aggressive care and broad forms of advance care planning. Normalization of palliative
420 care could make them more accessible to patients and families – but also offering a clear
421 framework for discussing palliative care to support healthcare providers who may be
422 uncertain about how to broach the subject. Specifically, improved visibility of palliative care
423 was noted as an important benefit, as many patients still remain unaware of the scope of
424 palliative care and what it can offer them:

# 425

426	*“Yes, exactly, that we, yes, that visibility, that people know it exists and where they can*

427	*possibly turn to and where it’s located. I think that can indeed be improved.” (Phase I*

428	*physician)*

# 429

430	A standardized approach could prevent difficult conversations from being delayed until crisis

431	moments, with improved expectation management being highlighted as an advantage,

432	especially with the only small chances of effectiveness in mind:

# 433

434	*“We still offer a trial option, so there’s still hope, but actually, that’s only a small hope. And*

435	*what if it goes wrong? Then we’re suddenly back at the point where there are no options. So*

436	*that can be anticipated.” (Oncologist)*

# 437

438	Enhanced interdisciplinary communication was also viewed as a benefit, with a more

439	integrated palliative care approach potentially ensuring that referring oncologists, GPs, and

440	phase I teams remained aligned. GPs specifically felt that they had a more active role to

441	play, yet were not given the opportunity to do so, as illustrated by the following quote:

# 442

443	*“I’ve experienced too often that patients die during the study. And where I then felt, darn, I*

444	*would have wanted to be involved, to tell the patient: don’t you see that what you’re doing is*

445	*pointless? Patients still die while receiving treatment.” (GP)*

# 446

447	Barriers to potential integration

# 448

449	Both patients and healthcare providers felt that bringing up palliative care while still receiving

450	treatment could be perceived as giving conflicting messages, undermining patients’ hope for

451	therapeutic benefit that in turn influences their wellbeing. This conflict was at the heart of

452	almost all interviews conducted:

# 453

454 *“I know that we are supposed to do advance care planning earlier, on paper, but just imagine*
455 *sitting in front of a patient to whom you are still offering a treatment option, and at the same*
456 *time, you have to say, ‘we should also start thinking about your end of life.’ That patient*
457 *wouldn’t understand what you’re doing, because essentially, you’re giving two contradictory*
458 *messages.” (Oncologist)*

# 459

460 Furthermore, phase I staff noted that integrating palliative care is complicated by the
461 uncertain prognosis of phase I trial participants, many of whom are still in relatively good
462 condition while in the trial, once again undermining the context necessary to start palliative
463 care conversations. Conflicts were also intrinsic to the phase I staff, as illustrated by the
464 following quote of a phase I physician who admits that despite the realization that trials have
465 a small chance of success, the balance between this fact and their therapeutic role is a
466 challenging one:

# 467

468	*“I think we’re too focused, that we let it appear as if it’s curative, too much still. I notice a*

469	*tension among our doctors as well – that’s what we struggle with as a team.” (Phase I nurse)*

# 470

471	The current lack of communication and continuity in treatment between these providers also

472	presents a barrier to this integration. Finally, healthcare providers noted hindrances by

473	resource constraints (e.g., limited time and insufficient staff) and by the current healthcare

474	system not financially incentivizing palliative care.

# 475

476	Strategies for potential integration

# 477

478 Healthcare providers proposed several specific strategies to facilitate the integration of
479 palliative care into phase I trials. These mainly included different suggestions for when
480 palliative care conversations could be systematically offered to patients within the trial
481 context. Some suggested scheduling a standardized palliative care conversation at trial
482 initiation with a palliative care specialist, although many participants pointed out that the
483 early stages of the trial are overloaded with information and that, for example, a month after
484 initiation would be better suited. Others felt that it had to be offered at the end of the trial, at
485 a minimum:

# 486

487	*“I think that upon ending phase I studies, regardless of whether it’s a decision by the patient*

488	*or exclusion due to medical study-related results, a conversation about palliative care and*

489	*end-of-life decisions should be offered.” (Palliative care specialist)*

# 490

491 Crucially, healthcare providers agreed that trajectories of patients, specifically with palliative
492 care in mind, are too diverse in terms of prognosis and condition to strictly adhere to a
493 structured approach – patients may not be open to such conversations. However, providers
494 agreed that while palliative care should not be pushed onto the patients, it should
495 nevertheless be more systematically brought up to note its potential benefits, with this
496 currently being a missed opportunity due to patients not receiving the information they may,
497 at some point, need:

# 498

499	*“I think you mainly have to offer [palliative care], that everyone should offer it a bit, and I*

500	*think it’s not bad if it’s also offered somewhat in phase I, I think those people are very much*

501	*in survival mode, and if that doesn’t work, then they fall off, and then, well, if they don’t know*

502	*that safety net of palliative care exists, that’s very unfortunate.” (Oncologist)*

# 503

504	Other suggestions focused on practical tools. The development of a protocol outlining when

505	and how palliative care should be introduced was suggested as a means to ensure

506	consistency across providers, as expressed succinctly by the following GP:

# 507

508	*“Protocoling isn’t just about rules. Through a protocol, you develop reflexes.” (GP)*

# 509

510 Providing patients with accessible information about palliative care resources and
511 incorporating quality-of-life monitoring tools, such as through standardized checklists and
512 through utilizing the questionnaires that patients already have to complete for study
513 purposes, were also recommended. Finally, electronic medical records were suggested as
514 the most feasible way to improve information exchange between oncologists, GPs, and
515 phase I staff, in language that is understandable for every healthcare provider and related to
516 palliative care discussions.

# 517

518	**Discussion**

# 519

520 This qualitative multiperspective interview study explored the current attention to quality of
521 life in care for phase I oncological trial participants, the current role of palliative care, and the
522 possibilities for better integrating palliative care in phase I oncological trials, from the
523 perspectives of patients, informal caregivers, and various healthcare providers. Our findings
524 indicate that palliative care is currently not systematically integrated into phase I trial settings
525 in Belgium, neither through proactive referral to palliative care specialists, palliative care, nor
526 through the consistent adoption of a generalist palliative care approach such as early
527 advance care planning conversations. While trial participants generally reported a positive
528 impact of participation on their quality of life, informal caregivers and healthcare providers
529 questioned whether the support offered was sufficiently holistic, often noting that quality of
530 life discussions remained limited to physical symptoms rather than general wellbeing. This
531 reflects a paradox: although all stakeholders recognized the importance of quality of life, it
532 often remained a reactive, and surface-level concern, rather than a fully integrated and
533 proactive focus within trial care.

# 534

535 For patients with advanced cancer, enrolling in a phase I trial often served as a lifeline. Many
536 participants described the trial as a source of hope and a means of staying in control – a
537 chance to ‘do something’ when standard treatment options were exhausted. This hopeful
538 mindset had emotional benefits: patients spoke of feeling optimistic simply by virtue of
539 having access to a new treatment. However, this hope came with considerable burdens.
540 Frequent hospital visits, stringent eligibility protocols, side effects, and the uncertainty of
541 unproven therapies all placed strain on patients and their families. Patients were willing to
542 endure these burdens as the price of maintaining hope. Within this context, the idea of
543 involving palliative care was often met with resistance or indifference from patients, but also
544 from healthcare providers, who feared that introducing palliative care discussions might
545 undermine patients’ hope. This mirrors broader findings that provider reluctance, rooted in
546 concerns about destroying hope, continues to delay timely palliative care conversations (29–
547 32). Yet, previous research on hope in the phase I context has called for an inclusion of hope
548 reframing within palliative care trajectories, shifting from supporting unrealistic hopes to
549 nurturing realistic hopes (33,34). Furthermore, palliative care interventions for patients
550 participating in phase I studies have been shown to alleviate distress and improve quality of
551 life for both patients and their caregivers, supporting the feasibility of integration of palliative
552 care in the phase I context, as opposed to the feared incompatibility (9,10,35).

# 553

554 Patients’ narrow view of palliative care as synonymous with terminal care is in line with
555 international research and reflects a significant gap in communication (36). Healthcare
556 providers reinforced the view of patients. They rarely initiated discussions about palliative
557 care, consequently also not providing patients with information about the benefits it may
558 provide. Although phase I staff noted that it is also their responsibility to discuss palliative
559 care, in practice, the topic was frequently sidestepped or deferred, with providers assuming
560 that someone else (such as a GP or the referring oncologist) would broach it at an
561 appropriate time. Moreover, fragmentation in the healthcare team exacerbated this issue.
562 Phase I trial specialists, referring oncologists, palliative care specialists, and GPs often
563 operated in silos with poor coordination. There was no clear consensus on whose role it was
564 to address advance care planning or introduce supportive care alongside the trial. This lack
565 of communication not only reinforced misconceptions but likely contributed to the resistance
566 patients displayed towards early palliative care engagement.

# 567

568 Informal caregivers and healthcare providers recognized important potential benefits of
569 introducing palliative care into the phase I setting: mainly, improved holistic care and the
570 normalization of advance care planning conversations before crises occur. Participants –
571 especially health care providers – proposed several promising strategies. A common
572 suggestion was to make palliative care discussions a routine (but flexible) part of the trial
573 care pathway. Rather than relying on individual providers to decide ad hoc when (or if) to
574 mention palliative care, the idea is to standardize certain touchpoints for these
575 conversations. These conversations could emphasize that services focused on quality of life
576 are available in parallel with the trial, framing it as an additional layer of support rather than
577 an alternative. Such an approach would ensure every patient is at least aware of palliative
578 care resources, even if they choose not to use them immediately. Education and culture
579 change were implicit in many of these strategies and has been suggested previously as an
580 important part towards the integration of palliative care in phase I settings, by normalizing
581 palliative care as a standard component of comprehensive cancer care (even in trials),
582 hoping to gradually erode the stigma and fear surrounding it (34). Referring oncologists also
583 have a pivotal role in this cultural shift. Their earlier introduction of palliative care – ideally at
584 the point of discussing referral to a phase I trial – can help set expectations and reduce
585 stigma, while making it easier for phase I staff to continue the conversation in a new care
586 context.

# 587

588 Our findings also highlight the missed opportunity to integrate quality of life questionnaires –
589 patient-reported outcome measures (PROMs) – meaningfully into clinical practice. In
590 contrast to phase III trials, where PROMs are often mandated, phase I staff members noted
591 that PROMs are rarely utilized in phase I trials, in line with recent international literature (37–
592 39). Even when collected, PROM data was typically solely collected in the context of
593 research purposes, leaving clinicians without systematic insight into patients’ evolving quality
594 of life needs. Strengthening PROM integration into clinical workflows could thus serve as an
595 important stepping stone toward more holistic care approaches in phase I settings (40,41).

# 596

597 Strengths of this study include the triangulation of perspectives across multiple stakeholder
598 groups, providing a nuanced understanding of both the barriers and opportunities for
599 integration. A main limitation of our study is that the sample included only patients actively
600 participating in phase I trials, potentially biasing findings towards more positive experiences
601 and towards lower openness to palliative care than might be seen in patients who declined
602 or discontinued participation. Additionally, selective recruitment to avoid patient burden may
603 have resulted in an overrepresentation of individuals in relatively good health. Furthermore,
604 our sample lacks diversity, with all participants being Dutch-speaking and predominantly
605 male.

# 606

607 Future research should explore and evaluate models for the feasible and sustainable early
608 integration of palliative care into phase I trial settings, based on our findings and previous
609 literature (10). Research has suggested future models of palliative care integration to rely
610 more on generalist palliative care delivered by oncologists or clinical trial coordinators, who
611 play a central role in the phase I trial process (42,43).

# 612

613 However, while previous palliative care interventions in the phase I setting have remained
614 confined to that context (10), our findings suggest that this is a necessary but likely not
615 sufficient step. It is also crucial to involve other healthcare providers who are part of the
616 patient’s care trajectory, particularly referring oncologists and general practitioners. These
617 professionals often have longstanding relationships with patients and may be best positioned
618 to provide appropriate palliative care and initiate sensitive conversations. Furthermore, the
619 systematic adoption of quality of life measures in the phase I trajectory may also be an
620 opportunity for increased visibility of care for quality of life and palliative care, as highlighted
621 by interviewed healthcare providers.

# 622

623 In conclusion, this qualitative study highlights that in current phase I oncological trials in
624 Belgium, a palliative care approach is not systematically integrated into patient care
625 trajectories. While the importance of quality of life was recognized in principle, it often
626 remained an afterthought to the primary mission of drug dosing. Healthcare providers and
627 informal caregivers acknowledged the potential benefits of integrating palliative care for
628 holistic support and advance care planning, even as patients expressed little openness to
629 the topic, associating it with terminal care. Although important barriers exist – particularly
630 around perceptions of incompatibility with active treatment and poor interdisciplinary
631 communication – healthcare providers identified strategies for systematic yet flexible
632 integration. These aim to ensure every patient is offered holistic support early and
633 consistently, while respecting individual readiness and preserving hope. Overall, these
634 findings underscore the need for structured, patient-centered approaches to support the
635 complex needs of patients participating in phase I trials.

# 636

637	**Acknowledgments**

638	We wish to thank Marthe Tulpin, Roselynn De Roeck, and Ruth Coussement for their help

639	with conducting interviews, as well as the study coordinators of the Drug Research Unit

640	Ghent and University Center for Clinical Research Antwerp for their help with data collection.

# 641

642 **References**
643 1.	Baker KM, Andersen L, McHugh M, Foxwell AM, Zhou Q, Ratcliffe SJ, et al. Regret in
644 Clinical Trial Participation Among Cancer Patients. J Palliat Med. 2025 Feb
645 1;28(2):185–92.
646 2.	van der Biessen DA, Oldenmenger WH, van der Helm PG, Klein D, Oomen-de Hoop
647 E, Mathijssen RH, et al. Self-reported quality of life and hope in phase-I trial
648 participants: An observational prospective cohort study. Eur J Cancer Care (Engl).
649 2018 Nov;27(6):e12908.
650 3.	Alouani E, Gazzah A, Mercier S, Bahleda R, Hollebecque A, Michot JM, et al. Profile
651 and outcome of cancer patients enrolled in contemporary phase I trials. Eur J Cancer.
652 2023 Jul;188:1–7.
653 4.	Ebata T, Shimizu T, Koyama T, Shimomura A, Iwasa S, Kondo S, et al. Improved
654 survival among patients enrolled in oncology phase 1 trials in recent decades. Cancer
655 Chemother Pharmacol. 2020 Feb 19;85(2):449–59.
656 5.	Wootten AC, Abbott JM, Siddons HM, Rosenthal MA, Costello AJ. A qualitative
657 assessment of the experience of participating in a cancer-related clinical trial.
658 Supportive Care in Cancer. 2011 Jan 4;19(1):49–55.
659 6.	Cassel JB, Del Fabbro E, Arkenau T, Higginson IJ, Hurst S, Jansen LA, et al. Phase I
660 Cancer Trials and Palliative Care: Antagonism, Irrelevance, or Synergy? J Pain
661 Symptom Manage. 2016 Sep;52(3):437–45.
662 7.	Hui D, Parsons H, Nguyen L, Palla SL, Yennurajalingam S, Kurzrock R, et al. Timing
663 of palliative care referral and symptom burden in phase 1 cancer patients. Cancer.
664 2010 Sep 15;116(18):4402–9.
665 8.	Finlay E, Lu HL, Henderson H, O’Dwyer PJ, Casarett DJ. Do phase 1 patients have
666 greater needs for palliative care compared with other cancer patients? Cancer. 2009
667 Jan 15;115(2):446–53.
668 9.	Ferrell B, Chung V, Hughes MT, Koczywas M, Azad NS, Ruel NH, et al. A Palliative
669 Care Intervention for Patients on Phase 1 Studies. J Palliat Med. 2021 Jun
670 1;24(6):846–56.
671 10.	Uhelski ACR, Wheless M, Davis EJ, Agarwal R. Palliative care integration for patients
672 on phase I cancer clinical trials. Curr Probl Cancer. 2023 Oct;47(5):101022.
673 11.	Bruera E, Hui D. Integrating Supportive and Palliative Care in the Trajectory of
674 Cancer: Establishing Goals and Models of Care. Journal of Clinical Oncology. 2010
675 Sep 1;28(25):4013–7.
676 12.	World Health Organization. Palliative care. https://[www.who.int/europe/news-](http://www.who.int/europe/news-)
677 room/fact-sheets/item/palliative-care. 2023.
678 13.	Temel JS, Greer JA, Muzikansky A, Gallagher ER, Admane S, Jackson VA, et al. Early
679 Palliative Care for Patients with Metastatic Non–Small-Cell Lung Cancer. New
680 England Journal of Medicine. 2010 Aug 19;363(8):733–42.
681 14.	Bakitas M, Lyons KD, Hegel MT, Balan S, Brokaw FC, Seville J, et al. Effects of a
682 Palliative Care Intervention on Clinical Outcomes in Patients With Advanced Cancer.
683 JAMA. 2009 Aug 19;302(7):741.
684 15.	Zimmermann C, Swami N, Krzyzanowska M, Hannon B, Leighl N, Oza A, et al. Early
685 palliative care for patients with advanced cancer: a cluster-randomised controlled trial.
686 The Lancet. 2014 May;383(9930):1721–30.
687 16.	Vanbutsele G, Van Belle S, Surmont V, De Laat M, Colman R, Eecloo K, et al. The
688 effect of early and systematic integration of palliative care in oncology on quality of life
689 and health care use near the end of life: A randomised controlled trial. Eur J Cancer.
690 2020 Jan;124:186–93.
691 17.	Hui D, Hannon BL, Zimmermann C, Bruera E. Improving patient and caregiver
692 outcomes in oncology: Team‐based, timely, and targeted palliative care. CA Cancer J
693 Clin. 2018 Sep 13;68(5):356–76.
694 18.	Horlait M, Chambaere K, Pardon K, Deliens L, Van Belle S. What are the barriers
695 faced by medical oncologists in initiating discussion of palliative care? A qualitative
696 study in Flanders, Belgium. Supportive Care in Cancer. 2016 Sep 16;24(9):3873–81.
697 19.	Vaismoradi M, Turunen H, Bondas T. Content analysis and thematic analysis:
698 Implications for conducting a qualitative descriptive study. Nurs Health Sci. 2013 Sep
699 11;15(3):398–405.
700 20.	Tong A, Sainsbury P, Craig J. Consolidated criteria for reporting qualitative research
701 (COREQ): a 32-item checklist for interviews and focus groups. International Journal
702 for Quality in Health Care. 2007 Sep 16;19(6):349–57.
703 21.	Lumivero. Nvivo. 2023.
704 22.	Sandelowski M, Barroso J. Classifying the Findings in Qualitative Studies. Qual

705	Health Res. 2003 Sep 1;13(7):905–23.

706	23.	Green J, Thorogood N. Analysing qualitative data. In: Silverman D, editor. Qualitative

707	Methods for Health Research. 1st ed. London: SAGE; 2004. p. 173–200.

708	24.	Stevens P, editor. Qualitative data analysis : key approaches. London: SAGE; 2022.

709	25.	Hsieh HF, Shannon SE. Three Approaches to Qualitative Content Analysis. Qual

710	Health Res. 2005 Nov 1;15(9):1277–88.

711 26.	Mayring P. Qualitative Content Analysis: A Step-by-Step Guide. SAGE; 2021.
712 27.	Burla L, Knierim B, Barth J, Liewald K, Duetz M, Abel T. From Text to Codings:
713 Intercoder Reliability Assessment in Qualitative Content Analysis. Nurs Res. 2008
714 Mar;57(2):113–7.
715 28.	Cohen J. A Coefficient of Agreement for Nominal Scales. Educ Psychol Meas. 1960

716	Apr 1;20(1):37–46.

717 29.	Clayton JM, Butow PN, Tattersall MHN. When and How to Initiate Discussion About
718 Prognosis and End-of-Life Issues with Terminally Ill Patients. J Pain Symptom
719 Manage. 2005 Aug;30(2):132–44.
720 30.	Almack K, Cox K, Moghaddam N, Pollock K, Seymour J. After you: conversations
721 between patients and healthcare professionals in planning for end of life care. BMC
722 Palliat Care. 2012 Dec 17;11(1):15.
723 31.	Pfeil TA, Laryionava K, Reiter-Theil S, Hiddemann W, Winkler EC. What Keeps
724 Oncologists From Addressing Palliative Care Early on With Incurable Cancer
725 Patients? An Active Stance Seems Key. Oncologist. 2015 Jan 1;20(1):56–61.
726 32.	Brighton LJ, Bristowe K. Communication in palliative care: talking about the end of
727 life, before the end of life. Postgrad Med J. 2016 Aug 1;92(1090):466–70.
728 33.	Yang GM, Ong WY, Tan J, Ding J, Ho S, Tan D, et al. Motivations and experiences of
729 patients with advanced cancer participating in Phase 1 clinical trials: A qualitative
730 study. Palliat Med. 2023 Feb 7;37(2):257–64.
731 34.	Escritt K, Mann M, Nelson A, Harrop E. Hope and meaning-making in phase 1
732 oncology trials: a systematic review and thematic synthesis of qualitative evidence on
733 patient-participant experiences. Trials. 2022 Dec 16;23(1):409.
734 35.	Treasure M, Daly B, Cao S, Fu P, Hong A, Weinstein E, et al. A randomized controlled
735 trial of structured palliative care versus standard supportive care for patients enrolled
736 in phase 1 clinical trials. Cancer Med. 2021 Jul 25;10(13):4312–21.
737 36.	Bellhouse S, Galvin L, Turner L, Taylor S, Davies M, Krebs M, et al. Phase I cancer
738 trials: a qualitative study of specialist palliative care. BMJ Support Palliat Care. 2020
739 Jun;10(2):234–41.
740 37.	Morga A, Dibenedetto S, Adiutori R, Su J. Patient-reported outcomes validated in
741 phase 3 clinical trials: a targeted literature review. Curr Med Res Opin. 2023 Jul
742 3;39(7):955–62.
743 38.	Byrom B, Everhart A, Cordero P, Garratt C, Meyer T. Leveraging Patient-Reported
744 Outcome Measures for Optimal Dose Selection in Early Phase Cancer Trials. JMIR
745 Cancer. 2025 Feb 28;11:e64611–e64611.
746 39.	Yap C, Lee Aiyegbusi O, Alger E, Basch E, Bell J, Bhatnagar V, et al. Advancing
747 patient-centric care: integrating patient reported outcomes for tolerability assessment
748 in early phase clinical trials – insights from an expert virtual roundtable.
749 EClinicalMedicine. 2024 Oct;76:102838.
750 40.	Haverman L, Luijten MAJ, Blackford AL, Absolom K, Basch EM, van Rossum MAJ, et
751 al. Truth and dare: patients dare to tell the truth when using PROMs in clinical
752 practice. Quality of Life Research. 2024 Dec 3;33(12):3299–307.
753 41.	Minvielle E, di Palma M, Mir O, Scotté F. The use of patient-reported outcomes
754 (PROs) in cancer care: a realistic strategy. Annals of Oncology. 2022 Apr;33(4):357–9.
755 42.	Fujiwara N, Ochiai R, Shirai Y, Saito Y, Nagamura F, Iwase S, et al. Qualitative
756 analysis of clinical research coordinators’ role in phase I cancer clinical trials.
757 Contemp Clin Trials Commun. 2017 Dec;8:156–61.
758 43.	Block SD, Billings JA. A need for scalable outpatient palliative care interventions. The

759	Lancet. 2014 May;383(9930):1699–700.

| 760 |  |
| --- | --- |
| 761 | **Supporting information** |
| 762 | S1 COREQ checklist. COREQ checklist. |
| 763 | S2 Topic guides. Topic guides. |

Figure 1	[Click here to access/download;Figure;Figure 1.png](https://www2.cloud.editorialmanager.com/pone/download.aspx?id=41228988&guid=fa1ef85b-5c7e-4071-93d6-e745b82c70b1&scheme=1)[<https://www2.cloud.editorialmanager.com/pone/download.aspx?id=41228988&guid=fa1ef85b-5c7e-4071-93d6-e745b82c70b1&scheme=1>](https://www2.cloud.editorialmanager.com/pone/download.aspx?id=41228988&guid=fa1ef85b-5c7e-4071-93d6-e745b82c70b1&scheme=1)

Supporting Information 1 - COREQ checklist

[Click here to access/download](https://www2.cloud.editorialmanager.com/pone/download.aspx?id=41228990&guid=94698018-25a7-4abb-9318-eb6a1955b3ed&scheme=1)

[**Supporting Information** S1 COREQ checklist.pdf](https://www2.cloud.editorialmanager.com/pone/download.aspx?id=41228990&guid=94698018-25a7-4abb-9318-eb6a1955b3ed&scheme=1)

Supporting Information 2 - Topic guides

[Click here to access/download](https://www2.cloud.editorialmanager.com/pone/download.aspx?id=41228992&guid=40757358-51ae-4ece-a45b-b5aacdc4df70&scheme=1)

[**Supporting Information** S2 Topic guides.docx](https://www2.cloud.editorialmanager.com/pone/download.aspx?id=41228992&guid=40757358-51ae-4ece-a45b-b5aacdc4df70&scheme=1)
