# Supplementary material for: Integration of palliative care into phase I oncology trials: A qualitative interview study with patients, informal caregivers, and healthcare providers
Source: PLoS One. 2026 May 5;21(5):e0347349. doi: 10.1371/journal.pone.0347349 (PMC13143114; doi:10.1371/journal.pone.0347349)
Supplement: Attachment — Submitted filename: Response_to_Reviewers_auresp_2.docx [file pone.0347349.s006.docx]

**RESPONSE TO REVIEWERS’ COMMENTS**

**MANUSCRIPT ID: PONE-D-25-38276**

**Integration of palliative care into phase I oncology trials: A qualitative interview study with patients, informal caregivers, and healthcare providers**

We thank the editor for the opportunity to respond to the remaining minor comments and submit a second revision of the manuscript. We also once again thank the reviewer for their helpful feedback to improve the manuscript. Below, we address the remaining comments raised – both the editor’s comments as noted in their email, as well as the comments added to the manuscript and the text they apply to.

All modifications to the manuscript described below have been made in the version titled ‘Revised Manuscript with Track Changes’.

A clean version without tracked changes has also been uploaded as a separate file labeled ‘Manuscript’. Please note that the noted locations of changes in the table below refer to the lines in the clean version of the Manuscript.

| Comment | Response | Location of changes |
| --- | --- | --- |
| 1. Kindly highlight the exact qualitative design that was employed. This should be made explicit under the study design section with appropriate references. | We clarified the qualitative design employed under the study design section with the appropriate reference, as follows:  “This study employed a qualitative descriptive research design using semi-structured face-to-face interviews (19).” | Lines 120-121 |
| 2. Ethical approval and considerations have been reported under "population and setting". Please provide a distinct section for this. | We moved the information on ethical approval from the Population and setting section to a new, distinct section ‘Ethical considerations’. | Lines 179-185 |
| 3. There is no section on trustworthiness or methodological rigour. This is central to qualitative research and must be included. Kindly create a specific section for this under the methods section. | We agree that trustworthiness and methodological rigor are central to qualitative research. In our study, we followed the Consolidated Criteria for Reporting Qualitative Research (COREQ) guidelines to ensure comprehensive and transparent reporting. Elements contributing to methodological rigor are integrated throughout the Method section, and focused on specifically in the Data analysis section. These include: (i) iterative data collection and analysis with regular team discussions and assessment of data saturation; (ii) systematic and collaborative development of the coding framework combining inductive and deductive approaches; (iii) independent double coding and iterative refinement of the coding scheme until a predetermined threshold of interrater reliability (.70 Cohen’s kappa) was reached; and (iv) member checking with participants from all stakeholder groups to validate and nuance the findings.  While it is not standard practice in our previous qualitative work to present these elements in a separate subsection, we appreciate the editor’s suggestion. However, to avoid redundancy, we have integrated these aspects within the existing sections. Should the editor consider a dedicated subsection on trustworthiness or methodological rigor essential, we would be happy to include this. | N/A |
| Consider to reduce the words/ sentences. Summarize the main objective. Avoid using  *(Abstract section: “To explore the perspectives of patients, informal caregivers, and healthcare providers on how quality of life and palliative care are currently addressed in phase I oncological trials, and to identify perceived benefits, barriers, and strategies for integrating palliative care.”)* | We summarized and rephrased the objective as follows to improve the readability: “To explore perspectives of patients, informal caregivers, and healthcare providers on quality of life and palliative care in phase I oncology trials, including perceived benefits, barriers, and integration strategies.” | Lines 30-32 |
| What was the timeframe for the study?  Has the study observed / maintained the ethical principles of research?  How about the sampling procedure?  Add: Ethical approval was gained from the relevant institutions  *(Abstract section: “We conducted a multi-perspective qualitative interview study across three Belgian university hospitals. The semi-structured interviews were analyzed using qualitative content analysis.”)* | We added the timeframe, sampling procedure, and ethical approval of the study to the abstract section as follows: “We conducted a multi-perspective qualitative interview study across three Belgian university hospitals from September 2022 to July 2024, using convenience and snowball sampling. The semi-structured interviews were analyzed using qualitative content analysis. Ethical approval was gained from the relevant institutions.” | Lines 33-36 |
| The sampling procedure is not very clear. Where exactly were the recruitments done & what process was followed to conveniently recruit participants? For instance, Ghent University Hospital is a big institution various departments? - please clarify  *(Method section: “Participants were recruited using convenience and snowball sampling. Recruitment started at Ghent University Hospital on September 1st 2022, with Antwerp University Hospital and UZ Brussel added as study sites on March 1st 2024. Recruitment ended at all sites on July 2nd 2024.”)* | We added the following details to the recruitment section: “Patients and informal caregivers were recruited from the dedicated oncological trial units at Ghent University Hospital and Antwerp University Hospital. Study coordinators introduced the study to patients and, when present, informal caregivers, and forwarded the contact details of interested individuals to the research team, who contacted them by telephone.” | Lines 153-156 |
| Apart from the informed consent , what are the other research ethics observed in this study ?  *(Method section: “All participants received study information and gave written*  *informed consent.”)* | We have moved this information to the new, distinct section ‘Ethical considerations’, including also the ethical approval process which was previously already mentioned under the Population and setting section, as follows: “This study was approved by the central ethics committees of Ghent University Hospital (6702022000253) and UZ Brussel (1432023000251) and by the local committee of Antwerp University Hospital”.  We furthermore added the following information: “Participants were informed they could withdraw at any moment and were also asked whether they agreed to be potentially contacted in the future for member checking.” | Lines 179-185 |
| This is not clear. Consider to shorten the sentences. Avoid very long sentences  *(Discussion section: “Our findings indicate that palliative care is currently not systematically integrated into phase I trial settings in Belgium, neither through proactive referral to palliative care specialists, palliative care, nor through the consistent adoption of a generalist palliative care approach such as early advance care planning conversations.”)* | We have rephrased this sentence as follows: “Our findings indicate that palliative care is not systematically integrated into phase I trial settings in Belgium. Patients are neither proactively referred to palliative care specialists nor supported through generalist approaches, such as early advance care planning conversations.” | Lines 524-527 |
| avoid ‘But flexible ‘ – consider language review  *(Discussion section: “A common suggestion was to make palliative care discussions a routine (but flexible) part of the trial care pathway.”)* | We have rephrased the sentence as follows: “A common suggestion was to make palliative care discussions a routine part of the trial care pathway, while allowing the necessary flexibility.” | Lines 571-573 |
| Clarify any conflict of interest in this study ?  *(Discussion section: Conclusion paragraph)* | We previously described in the mandatory ‘Competing Interests’ section that “The authors have declared that no competing interests exist.” As this will be published alongside the article, we have not added it in the discussion section to avoid redundancy. | N/A |
| ‘Although’ – is not started in new sentence.  Consider review language proofreading of your study  *(Discussion section: “Although important barriers exist – particularly around perceptions of incompatibility with active treatment and poor interdisciplinary communication – healthcare providers identified strategies for systematic yet flexible integration.”)* | We have rephrased the sentence as follows: “Important barriers exist, particularly around perceptions of incompatibility with active treatment and poor interdisciplinary communication, yet healthcare providers identified strategies for systematic yet flexible integration.” | Lines 627-629 |
